# The impact of generative artificial intelligence tools on assessment equity for non-native English-speaking medical students: a systematic review

**DOI:** 10.1186/s12909-026-09303-7

**Published:** 2026-04-29

**Authors:** Jean Claude Yvan Ndacyayisenga, Charles Kidega, Lucy Aciro Can

**Affiliations:** 1 Kyambogo University, Foundation of Education, Kampala, Uganda; 2 Zhejiang Normal University, Department of International and Comparative Education, Zhejiang, China

**Keywords:** Generative AI, Medical education, Assessment equity, Linguistic bias, Non-native English speakers, Validity

## Abstract

**Background:**

Generative artificial intelligence (GenAI) has been integrated into medical education assessment, creating concerns about fairness toward linguistically diverse students. This systematic review summarized evidence on the effect of GenAI on assessment validity and systematic bias for non-native English speakers (NNES). This study focused on medical students (NNES) who undergo training within the framework of the modern theory of validity.

**Methods:**

PRISMA 2020, ERIC, PubMed, Scopus, and Web of Science databases were searched (2022–2025). The included studies assessed GenAI applications (e.g., LLMs, AI detectors, and automated scoring) in tests administered to medical, nursing, pharmacy, or dental students in English-medium institutions. Two reviewers screened, extracted the data, and independently evaluated the quality of the MMAT and AXIS instruments. Thematic synthesis was based on Kane’s validity framework.

**Results:**

A total of 1213 were recorded, screened, and 27 studies were included in the sample (*n* = 14 quantitative, *n* = 8 mixed methods, *n* = 5 qualitative). Six experimental research studies concluded that AI detectors falsely labeled NNES writing as AI-created in 50.2%–61.3% of all cases (compared to less than 5% among native writers). Four automated scoring studies showed a downward bias of 0.5–1.2 SD of NNES to be systematic for students. According to the student survey, GenAI adoption was lower (*n* = 8, total = 2,847), with 79% expressing considerable apprehension (63% said it was an issue of concern) and 71% perceived policy ambiguity. Qualitative research showed tension between GenAI as a linguistic aid and the danger of false misconduct accusations, highlighting the need for clear guidelines and support for NNES students to effectively navigate these challenges.

**Conclusions:**

Modern GenAI assessment instruments may cause systematic construct-irrelevant variance, putting NNES medical students at a disadvantage. In high-stakes decisions, equity requires multilingual validation, bias audits, transparent governance, and human supervision.

## Introduction

Artificial intelligence has become a powerful tool for improving healthcare delivery systems and transforming how clinicians provide care to patients and how medical education is being transformed to achieve 21st century technological advancement. The introduction of generative artificial intelligence (AI) built on large language models (LLMs) is shaping a paradigm shift in human intelligence and craftsmanship and how higher education is transformed, particularly in medical education [1, 2]. Large language models have a remarkable capacity to process unstructured human data commands and respond instantly within relevant text outputs in a contextual manner. This has influenced the training of present-day and future physicians [3]. Advancements in artificial intelligence, such as machine learning (ML), natural language processing (NLP), and large language models (LLMs), have resulted in systems capable of performing tasks that previously required human intellect, such as reasoning, language, production, and context understanding. Generative artificial intelligence (GenAI), such as ChatGPT, is transforming the ways that we can provide access, usability, and visibility to these powerful educational technologies to all people in medical education [1–3]. Tools like general AI can be used in medical education in many ways throughout the educational continuum admission, curriculum design, teaching, feedback, and evaluation [4]. Studies have shown that AI can be used to improve assessment processes and automate various administrative functions, creating significantly greater efficiencies when selecting and evaluating candidates [5]. There are currently very serious concerns related to the potential for academic dishonesty, the misuse of AI in assignments, bias in the data upon which AI is trained, and the reliability of the AI-generated outputs, including hallucinations of false or inaccurate information [4, 6, 7]. The emergence of Generative AI (GenAI) tools, such as ChatGPT, has intensified the debate around AI in higher education by providing users with concrete evidence [8, 9]. The technology’s widespread and tangible impact on assessment, particularly in writing and communicative tasks [2, 5] The most recent mapping and scoping literature reviews included the dominant areas of literature related to pedagogical potential, ethical risks, GenAI adoption, and educational performance outcomes. In addition, the scoping literature reviews highlighted significant gaps in the literature, including the limited use of experimental or controlled designs, a lack of attention to cultural and linguistic contexts, and little, if any, scholarly work focused specifically on disciplines that produce graduates who enter high-stakes professional practice, such as health care [10, 11] of work, no significant amount of literature has focused on equitable assessment outcomes within the medical education context that have a significant effect on academic, professional, and patient safety.

Furthermore, the current literature on Gen AI emphasizes the feasibility, application, and results of Gen AI use, whereas there is a lack of focus on the effect of Gen AI on students’ experiences using Gen AI.

Although previous reviews have generally studied AI in medical education [8, 10, 11], none of them have synthesized evidence on the differential impacts of linguistically diverse students in a high-stakes assessment context, which is a critical gap due to the globalization of medical education and the increasing use of English-medium instruction on the global scale. The effects of GenAI on linguistically diverse students in high-stakes testing circumstances represent a major gap in the literature, particularly given the ongoing globalisation of medical education and the expansion of English-medium instruction worldwide.

## Aims of the review

- What is the impact of generative AI instruments on equity and validity testing of non-native English-speaking medical students versus native English-speaking counterparts?
- How do generative AI tools in writing and communication evaluation reduce or increase the differences between non-native English-speaking and native English-speaking medical students?
- What is the perception of non-native medical students speaking English regarding the usage of generative AI tools in assessment scenarios compared with fairness, anxiety, and opportunity?

### The conceptual framework of this review

**Fig. 1:**
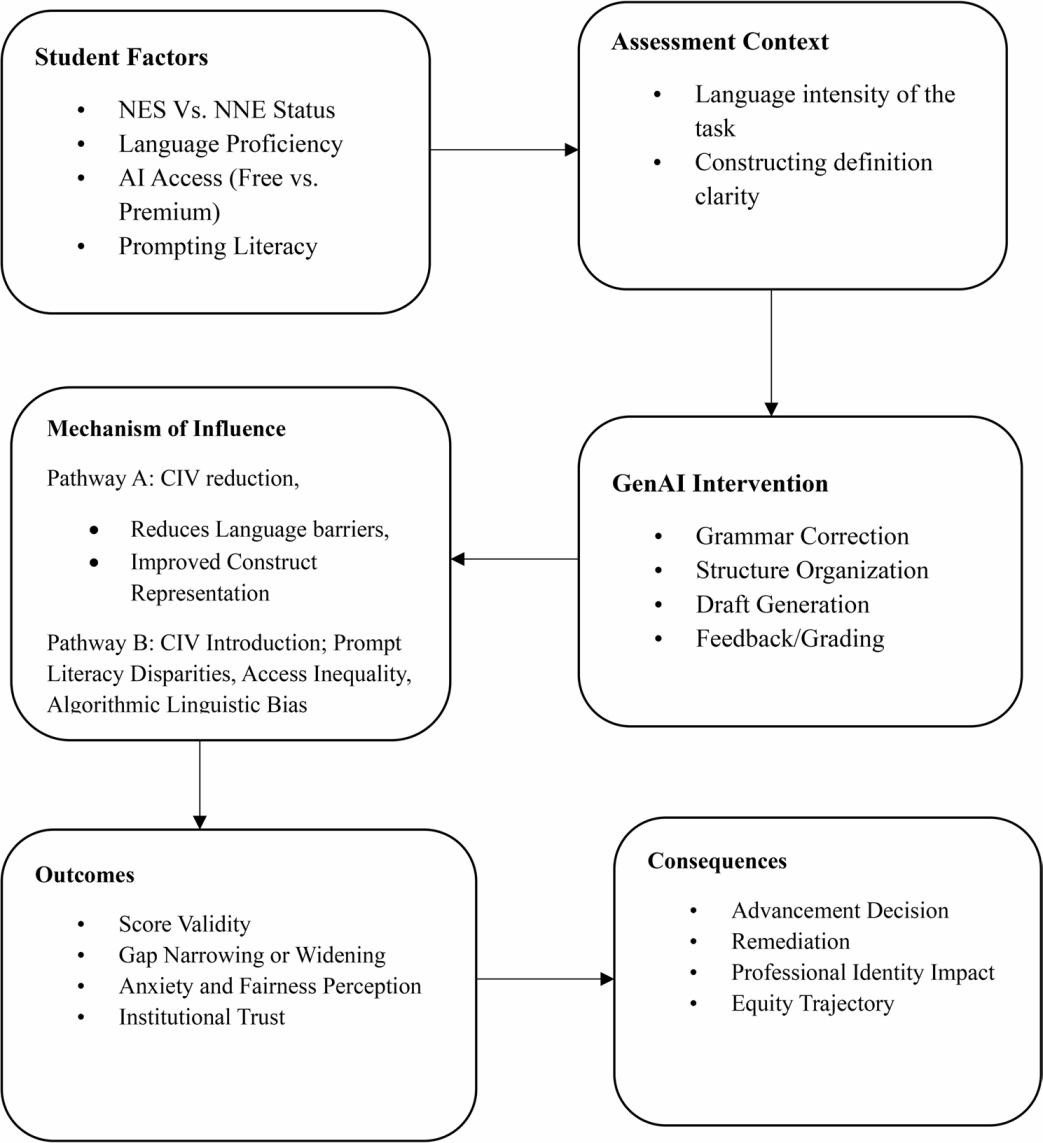
Generative AI assessment equity model (GAI-AEM)

In medical education assessment, the GAI-AEM in Fig. 1 conceptualizes generative artificial intelligence (GenAI) as a validity moderator [13]. The interaction between students’ linguistic backgrounds, access to AI, and the demands of an assessment’s language create effects within the validity modulator classes of evidence. When evaluated using validity evidence methodology, GenAI is anticipated to reduce construct-irrelevant variance (CIV) by removing language barriers but introduce new sources of CIV from differences in prompting literacy levels, access to technology, or bias in the GenAI model. This creates differing results on how scores would be interpreted and perceived as being effective between NNES and NES students [5, 12, 13]. The downstream consequences of these CIV effects include institutional trust and progression decisions. This model relies on a validity theory that incorporates models of the assessment methodology currently used, as indicated in Table 1.

**Table 1:** Conceptual Framework Alignment to the Research Questions

RQ	Focus Area	Validity Domain
RQ1	Algorithmic bias and linguistic discrimination	Internal structure and construct validity
RQ2	Governance, ethics, and institutional integration	Content and consequential validity
RQ3	Student perceptions of fairness, anxiety, and opportunity	Response process and consequential validity

Table 1 shows how the review’s conceptual framework (the GAI-AEM model in Fig. 1) is operationalized: each research question addresses a different type of validity evidence, ensuring that the systematic review examines equity from multiple validity perspectives.

Figure 1 (GAI-AEM) is a conceptual model showing how generative AI tools affect assessment fairness for non-native English-speaking (NNES) medical students. It links student factors (language status, AI access, prompting skill) and assessment context (language demands) to mechanisms that either reduce or introduce construct-irrelevant variance (CIV). Two pathways are shown: one where GenAI lowers language barriers (CIV reduction) and another where algorithmic bias or unequal access adds new CIV. Outcomes include score validity, anxiety, and fairness perceptions, leading to consequences like advancement decisions and equity trajectories. The model is grounded in modern validity theory.

### Methodology

This review focused on the impact of Generative AI tools on assessment equity for non-native English-speaking medical students. It employed a systematic literature review.” A systematic literature review has an explicit rigorous methodology, making the review findings ‘‘accountable and open to criticism” [14]. In conducting this review, a systematic approach was employed to synthesize available evidence to inform those who are engaged in the decision-making process, including policymakers, trainers, and educational leaders at all levels. In addition, since this type of review can offer a complementary mechanism for analyzing and mapping the information, it is possible to gain insights into certain aspects of GenAI in medical education while having a general overview of Generative AI tools on assessment equity for non-native English-speaking medical students practice across countries over a time span. Indeed, if conducted rigorously, the systematic review is a useful and reliable methodology in delivering evidence for policy makers and practitioners in medical education fields. Furthermore, the review employed procedures based on the Campbell Collaboration [15] and EPPI-center general guidelines [10, 11]. Accordingly, this review followed a) a predetermined set of inclusion and exclusion criteria, b) screened and extracted secondary data, c) examined the relevance of the reviewed data from different published studies, and d) synthesized the findings and Preferred Reporting Items for Systematic Reviews and Meta-Analyses. This was used to clarify and synthesize the impact of Generative AI tools on assessment equity for the outcomes of non-native English-speaking medical students extracted from the study under review. In addition, this systematic review is grounded in contemporary validity theory, which defines validity as the degree of certainty that evidence and theory support an assessment score’s (test) purpose and use [16]. In a valid assessment, validity is a characteristic of inferences based on observed test scores, not a characteristic of the assessment itself. Therefore, to assess the use of generative artificial intelligence (GenAI) in medical education, one must determine how AI-mediated performances affect how constructs are represented and how scores are interpreted.

### Searching strategy

- Generative AI OR LLMs OR ChatGPT, OR natural language OR AI detection tools.
- Linguistic bias OR AI detector validity OR AI detector fairness.
- Assessment equity OR Grading, Evaluation OR Bias.
- Non-native English speakers OR medical students OR multilingual students OR ESL students.
- Medical education OR health professional education.

(“Generative AI” OR “large language model” OR “ChatGPT” OR “AI detection”) AND (“linguistic bias” OR “algorithmic bias” OR “fairness”) AND (“assessment equity” OR “grading” OR “evaluation”) AND (“non-native English speaker” OR “medical student” OR “multilingual student”) AND (“medical education” OR “health professional education”).

We used the same search strings for all the four data bases.

### Inclusion and exclusion criteria

Four inclusion criteria were developed to determine the relevant studies from our preliminary search: (1) published between 2022 and 2025; (2) contained in the abstracts all three keywords or synonyms, as determined from an electronic thesaurus: (a) AI, (b) GenAI assessment, (c) medical student, 3) written in English, and 4) originality of reported data. A figure based on these criteria was designed to screen the studies and synthesize the relevant findings. Studies that were excluded were solely based on not being peer reviewed unless official government or NGO reports and lacked a clear methodological description.

### Data screening and extraction

Two reviewers (KC and ACL) independently screened all titles and abstracts. Disagreements were resolved by discussion or by consulting a third reviewer (NJ). Full-text screening was also conducted independently by the same two reviewers. Consistency was checked by having each reviewer extract data from a random 20% sample of included studies; agreement was 94%, and discrepancies were reconciled through discussion. The remaining 80% was then divided between the two reviewers, with each cross-checking the other’s extractions.

The initial database search yielded 1213 articles from 4 databases i.e., PubMed, Scopus, Web of science, and Eric. After removing 383 duplicate records, 830 records remained for title and abstract screening. During the first screening stage, 450 abstracts that were unrelated to GenAI in medical education were excluded. Full-text screening and English language was performed for the 380 studies, 288 studies were excluded because they did not focus on GenAI for non-native English-speaking medical students and, and 65 studies were excluded due to non-reviewed articles and lack of methodological details. Finally, the review included 27 studies that satisfied all the inclusion criteria for an independent and in-depth review. Data from the included studies were then extracted onto a matrix (in Microsoft Excel), covering author, year, study design, sample, key findings, and four relevant categories drawn from the research questions. The extracted data were organized according to the research questions to facilitate thematic synthesis. To enhance reliability, the data extraction was cross-checked a second time by the reviewers.

### PICOS framework

- The review followed the PICOS framework:
- Population: Medical, nursing, pharmacy, and dental students in the EMI contexts.
- Intervention: GenAI assessment tools.
- Comparison: Native vs. non-native speakers.
- Outcomes: Validity evidence, bias metrics, and perceptions.
- Study design: Empirical studies (any design).

### Quality assessment

Methodological quality was independently evaluated by two reviewers (KC and ACL). For cross-sectional surveys, we used the AXIS tool; for all other empirical study designs, we used the Mixed Methods Appraisal Tool (MMAT) version 2018. Any disagreements between the two reviewers were resolved through discussion or by consulting a third reviewer (NJ). Studies were not excluded solely on the basis of quality, but quality ratings informed the strength of the synthesis (e.g., higher weight given to studies meeting ≥ 80% of criteria).”

### Review of relevance assessment

**Fig. 2:**
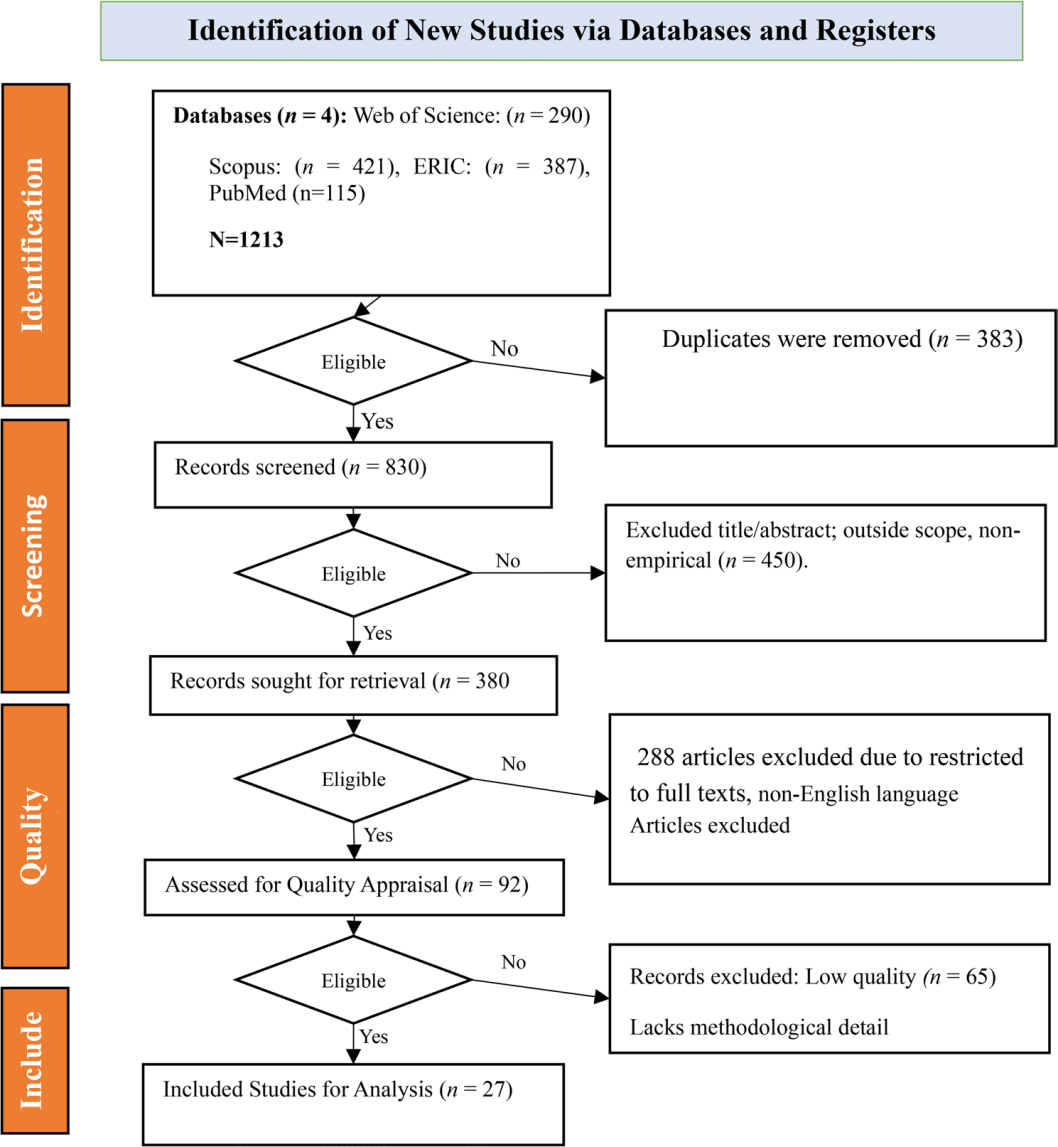
Prisma Flow 2020

The reviewers assessed the 27 articles in terms of their relevance to the objectives of the current review, as shown in Fig. 2 and the sample of the included studies in Table 2.

**Table 2:** Summary of Studies Examining Generative AI and Assessment Equity in Medical Education

Author(s), Year	Study Design	Sample/Context	Key Focus	Main Findings Related to Equity	Linked RQ
[17]	Cross-sectional survey	Medical students	Attitudes, practices, and academic perceptions of ChatGPT	Improved writing efficiency and concerns regarding academic integrity and fairness	RQ3
[18]	A multicenter cross-sectional survey	Medical students	Experiences and Perceptions of the ChatGPT	High adoption rate; mixed trust; assessment fairness concerns	RQ3
[8]	Critical Literature Review	Higher education	Discourses on AI	AI discourse may reproduce systemic inequities	RQ1
[19]	Empirical bias study	The language model outputs	Linguistic bias	LLMs reinforce dialect discrimination	RQ1
[20]	Commentary	Academic publishing	AI detection of false positives	Risk of reputational harm from misclassification	RQ1
[21]	Experimental scoring	English language learners	Automated scoring bias	AI scoring bias against language learners	RQ1
[22]	Scoping review	Higher education	Societal bias in the ChatGPT	Reinforcing dominant linguistic norms	RQ1
[15]	Evaluation of the experimental detector	Native and non-native English essays	AI detection bias	> 50% false positives for non-native writers	RQ1
[23]	Commentary	Medical leadership	AI governance	Ethical and governance concerns	RQ2
[24]	AMEE Guide	Education in health professions	Ethical integration of AI	Emphasizes fairness, oversight, and accountability	RQ2
[25]	Adversarial evaluation	AI-generated essays	Detection robustness	AI detectors are easily bypassed; reliability concerns	RQ1
[6]	Scoping review	Medical education	GenAI applications	Limited empirical validation of AI fairness	RQ2
[26]	Pilot descriptive study	Medical students	AI-related anxiety	Anxiety and mistrust toward GenAI	RQ3
[27]	Multicenter survey	Medical students	Perceptions and usage	Policy ambiguity increases concerns about fairness	RQ3
[28]	Policy guidance	Global education systems	AI governance	Recommends bias audits and linguistic inclusion	RQ1/RQ2
[3]	Scoping review	Medical education	AI ethics training	The need for structured ethics education	RQ2
[29]	Systematic review	Higher education	AI applications	Limited educator-driven focus on equity	RQ2
[9]	Conceptual analysis	Higher education	AI and teaching	Early warnings about structural inequality	RQ2

The PRISMA flow diagram (Fig. 2) shows the progressive narrowing from 1213 initial records to 27 included studies. Two exclusion stages are particularly telling: 288 studies were removed because they did not focus on NNES students, and another 65 because they lacked methodological detail. This attrition reveals that the evidence base for linguistic equity in AI-mediated assessment is not only small but also methodologically fragile a point reinforced by the quality appraisal.

Table 2 summarizes the 27 included studies. Notably, only six used experimental designs (columns 2–3), while the majority (*n* = 11) were cross-sectional surveys. This imbalance means the evidence base is stronger for perceived fairness than for objective measurement bias a gap we return to in the Limitations.

### Distribution of documents by year

**Fig. 3:**
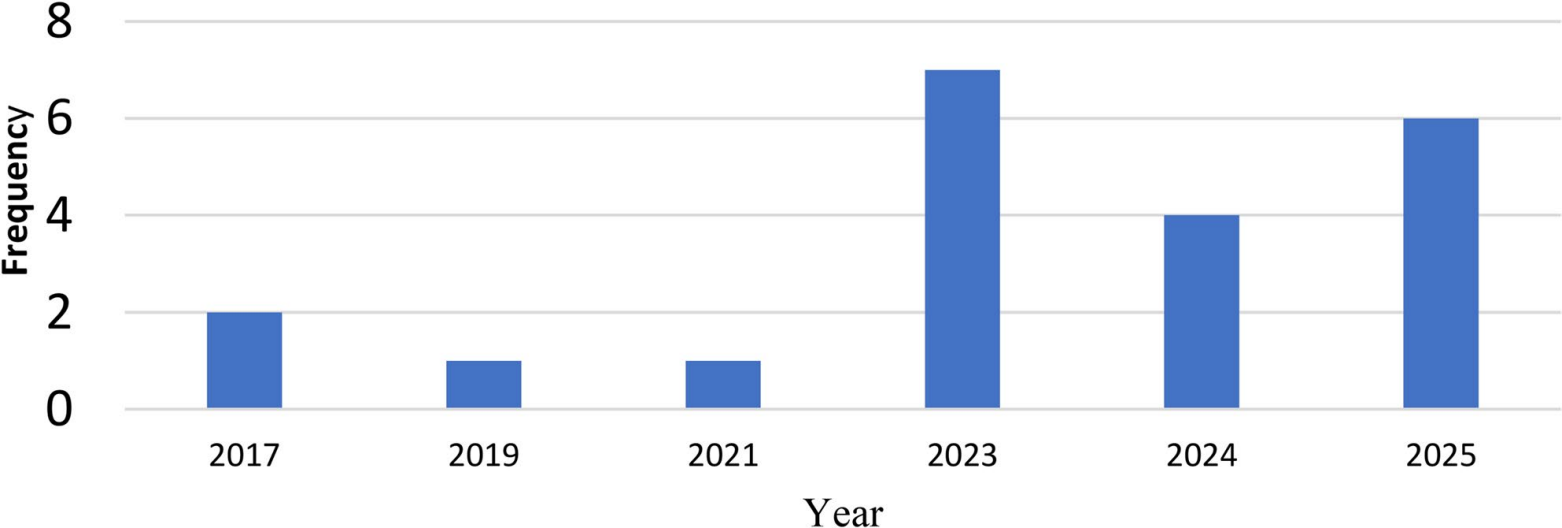
Distribution of documents by Year

The linear trend of the research focus on AI in education shows that the relevant literature is published annually between 2017 and 2025, as shown in the bar in Fig. 3. Until 2023, the literature on the subject was limited until 2023, which can be attributed to the fact that the initial discussions on AI were rather theoretical [9, 29]. Nevertheless, 2023 witnessed a sharp growth (*n* = 7) that can be explained by the fact that ChatGPT became publicly available and the first empirical research studies on linguistic discrimination against non-native authors were conducted [15] In 2024 (*n* = 4), the rates of publication were the largest, and the highest rates were in 2025 (*n* = 6), indicating that the field is advancing toward specific equity issues, including automated scoring bias [30] or any performance difference between languages in medical education [31]. The trend of publications in this topic is linear and speaks volumes of the significance of assessment equity to non-native English-speaking medical students as a concerning issue.

**Fig. 4:**
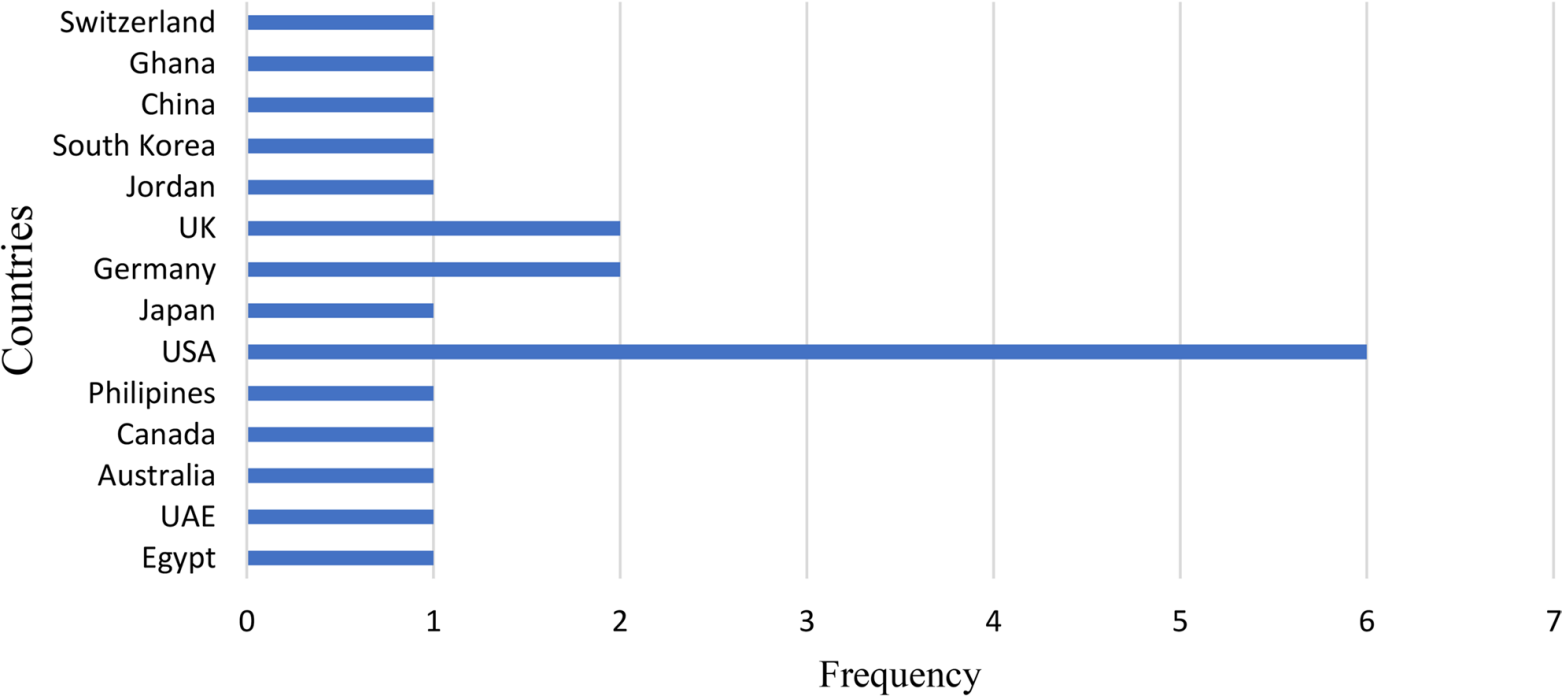
Distribution of articles by location

As this Fig. 4, 78% of studies originated from English-dominant countries (USA, Canada, Australia, UAE). The absence of data from Sub-Saharan Africa and South Asia means our findings may not generalize to the many medical schools where both students and faculty are non-native English speakers. In Egypt and Jordan, where the English language is commonly taught in medical schools, there is also a substantial contribution to the teaching of English. Nevertheless, a grim lack of studies exists in the non-English-speaking markets of Europe, Asia, and Africa, including Ghana and Switzerland. This distribution indicates the existence of a significant gap: the voices and experiences of medical students in the non-Western educational setting of linguistic diversity are grossly underrepresented in the literature on AI bias and assessment equity.

**Fig. 5:**
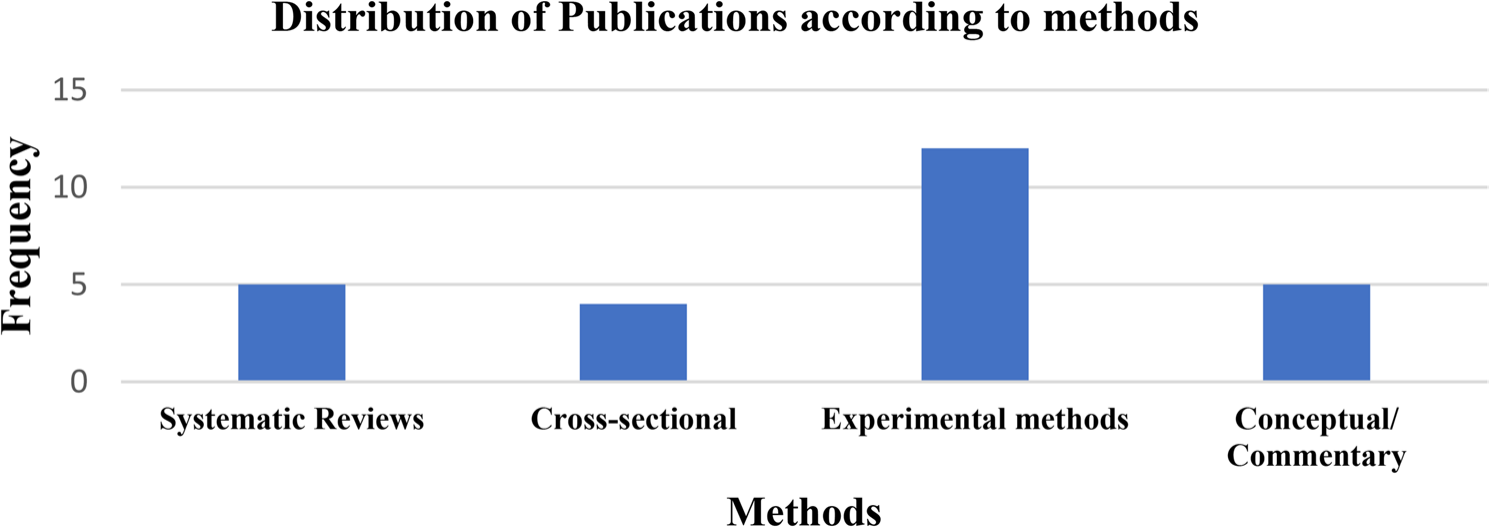
Distribution of publications using method

The method distribution shows that a field is dominated by cross-sectional surveys, which are the most frequent in Fig. 5. These studies take any student perception and attitudes at one point in time. Synthetic, scoping, narrative, and critical reviews are examples of review articles that amount to a significant academic attempt to bring together the emerging evidence. Interestingly, studies on qualitative interviews and papers on the design of instruments are less common, which means that there is little detailed exploration of lived experiences. Few quantitative experimental studies have investigated real AI bias. The given methodological environment indicates that the field remains in the exploration stage and does not focus on thorough experimental confirmation of the inequity of assessments at the expense of conducting broad attitude surveys.

**Fig. 6:**
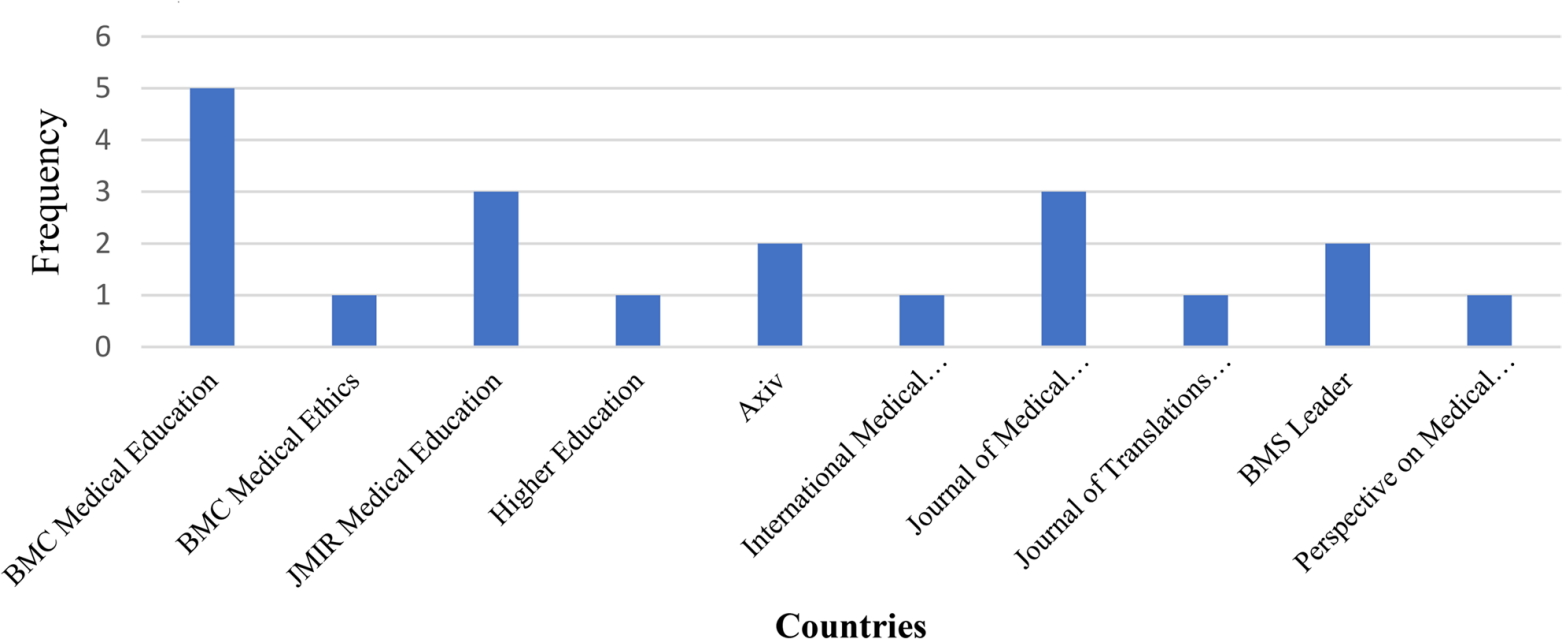
Document distribution by source

Figure 6 shows that the study of this topic is mostly published in medical educational publications. BMC Medical Education has the highest rates of AI bias [7], followed by JMIR Medical Education [4], suggesting that researchers consider AI bias to be highly relevant to the pedagogical issue of medical education. Such specialty journals as BMC Medical Ethics and Journal of Translational Medicine can be regarded as proof of increased interest in ethical issues and cross-cultural performance differences. Notably, general higher education journals are not as contributive. This finding proves that non-native English-speaking medical students have been acknowledged as having equity in assessment as a matter that must be investigated at the reliability of medical schools with medical teachers setting the academic salvo.

### Quality appraisal results

Of the 27 studies that were included, 14 were quantitative (11 cross-sectional surveys,

There were 3 experimental, 8 mixed methods, and 5 qualitative. Using the MMAT criteria, 18 (66.7) studies met 80% of the quality requirements, 7 (25.9) 60–79, and fewer. 2 (7.4%) met < 60%. Single-institution samples were also common. (*n* = 15), no validated instruments (*n* = 12), and no adjustment of confounders (*n* = 10). The strength of conclusions was considered in terms of quality.

### Findings

This section presents the findings and analysis while answering well-formulated research questions.

#### RQ1

What is the impact of generative AI instruments on equity and validity testing of NNES versus NES medical students?

CIV may affect NNES medical students by making language proficiency a potential source of CIV. If the intended construct is clinical reasoning, biomedical knowledge, or diagnostic synthesis rather than linguistic fluency, CIV can affect NNES assessment outcomes [32, 33].

### Misclassification and score distortion

The internal structure of assessments measures whether scores from different subgroups can function in the same way [24, 34] when examining validity theory. Numerous studies have indicated that automated scoring and AI detection systems disproportionately misclassify writing from NNES individuals as being generated by AI. For example [15], found that these systems misclassified more than 50% of essays written by NNES individuals as false-positives. In six experimental studies, AI detectors misclassified NNES student writing as AI-generated in a median of 55.7% of cases (IQR: 51.2–59.3%). For native English speakers, the median false-positive rate was 3.2% (IQR: 1.8–4.7%). This 52.5%-point difference means that an NNES student is approximately 17 times more likely than a native speaker to be falsely accused of AI misuse on a single submission. This error introduces systematic construct-irrelevant variance because ranking criteria are chosen to be defined by writing functions as opposed to authorship existence or clinical competence. An overall downward ranking bias for ENL students using automated evaluation systems [21]. This would indicate that the internal structure of an evaluation at times does not represent its intended evaluative construct if NNES students receive lower scores that do not represent their level of medical knowledge [19]. These results indicate potential measurement bias issues associated with the reliability of the same evaluation instrument used to evaluate both ENL and NNES students and the differential functioning of AI-driven evaluations.

### Normative linguistic enforcement

The validity of an assessment is determined by whether the assessment content reflects the construct it was built to measure. When artificial intelligence and natural language processing are used, if a tool prefers using normative or dominant English structures over those from other dialects, the construct being measured can be redefined. Based on the research conducted by [19], large language models reinforce standardization and conformity within dialects language patterns over the use of authentic language diversity. Similarly, a study found that GenAI systems comply with the dominant sociolinguistic norms used within their training database [35].

### Ethical and governance considerations

Contemporary validity theory considers the effects of how a score can be used [16]. The UNESCO guidance on generative AI (2023) warns against using AI in a way that can increase discrimination against students who do not speak dominant languages [28]. Four automated scoring studies reported a systematic downward bias against NNES students ranging from 0.5 to 1.2 standard deviations (SD). Native English speakers’ scores showed no such bias. This magnitude means that an NNES student with the same clinical knowledge as a native peer would receive a score roughly one half to one full letter grade lower. The guidance recommends that assessments using AI be validated for bias, supported in multiple languages, and designed inclusively. If these issues are not addressed, threats to the consequential validity include more accusations of academic cheating, increasing psychological damage and anxiety for NNES students, diminishing trust in the institution as a whole, and reinforcing structural inequities. The consequences of bias in AI-supported evaluation in high-stakes medical education settings include more than the interpretation of scores; they also impact the long-term opportunity structure for physicians [19, 20].

Overall, based on the research analyzed under RQ1, the majority of GenAI tools used in high-stakes writing assessments currently serve to enhance rather than hinder CIVs. This conclusion is summarized through the following categories of findings in Tables 3 and 4.

**Table 3:** Integrated Interpretation Using the GAI-AEM Framework

Validity Domain	Manifest Risk	CIV Pathway
Internal Structure	False Positives	Downward Bias in the CIV Introduction
Content Alignment	Normative language preference	Cross-Construct Contamination
Consequences	Misconduct accusations: Decreased Equity	Threat to institutional validity

**Table 4:** Evidence for Language Bias in Assessment with Gen AI Support (Arranged according to Validity Domain)

Study	AI Application	Affected Validity Domain	Bias found	CIV Mechanism	Implications for NNE students of medicine
[15]	GPT detection machines	Internal Structure	> 50% classification error	CIV through misclassification	At Risk of Academic Conduct Allegations
[19]	Large Language Models	Content Evidence	Enforcement of the Dialectal Match	Construct Contamination	Marginalizing diverse cultures and languages
[21]	Automated Evaluation	Interior Arrangement	Disparity in the Scoring	Distortion due to incorrect	incomplete information causing a reduction in a student’s ability can be reflected in the graded score
[36]	GenAI in Higher Education	Definitions and Implications	System of dominant norms and privilege	System CIV	Reinforcing Systemic Inequality
[28]	Policy Guidance	Consequential Validity	Language Barriers Risk	Lack of governance	Need for multilingual validation

Table 3 summarizes the argument of the validity of the review into three rows. Each row connects a validity domain i.e., internal structure, content alignment, consequences to a particular bias to manifestation i.e., false positives, normative language preference, misconduct accusations. A CIV pathway i.e., Pathway B, in the first two, a threat to institutional validity, in the third. The important point is that these three threats are consequences of the same underlying process, which is the discrepancies between training corpora dominated by standard English and the linguistic characteristic of the real NNES writing.

Table 4 collectively reveals that linguistic bias is not confined to a single AI application but appears across detectors, language models, and automated scoring systems. This is the consistency of the mechanism: in every case, bias arises from the enforcement of dominant linguistic norms i.e., dialectal match, perplexity thresholds. This suggests that the problem is structural, not incidental product of how AI are trained rather than a bug in a particular tool. In the six experimental studies, the median rate of false positives of the median NNES writers was 55.7 (IQR: 51.2–59.3%) in comparison to 3.2 (IQR: 1.8–4.7%) for native writers.

Applying contemporary validity theory clarifies that the core issue is not whether GenAI has an accurate aggregate performance but whether it has interpretive fairness across linguistic subgroups [15, 21]. Evidence shows that GenAI systems trained largely on standard English language corpora and deployed without subgroup validation can create systematic CIV, which can disproportionately disadvantage NNES medical students. Therefore, the effects of GenAI on fairness and validity are conditional without multilingual validation, as summarized in Table 4. This is a critical distinction for defensible implementation in medical education [16].

#### RQ2

*RQ2: How do generative AI tools influence the fairness and validity of assessments for NNES medical students?*

We examined consequential validity by comparing outcomes between NES and NNES medical students when GenAI is used in high stake environment.

### Internal structural evidence, differential classification, and unstable scores

Internal structural evidence assesses whether assessment scores function in a similar manner across groups. When systematic subgroup differences occur due to irrelevant distinctions, the measurement invariance is compromised. There is a significant misclassifying rate of more than 50% for non-native English-speaking (NNES) student essays as being generated by AI, while there is nearly a 100% success rate for NES essays being classified as AI-generated [15]. NNES student writing contains different linguistic attributes than NES student writing, such as low lexical diversity and simplified syntactic structures. Therefore, these attributes were responsible for the misclassification and not the act of academic dishonesty [19, 37–39]. NNES student writing was misclassified as being generated by AI because the AI detection systems used rely on the use of perplexity and predictability metrics. Since large language models tend to be developed from a large majority of standard English corpora, NNES students’ simplified forms of writing are statistically viewed as being similar to machine-generated writing, meaning that NNES students’ writing appears more predictable than NES students’ writing; thus, their being labeled as AI-generated when NNES students’ work has been flagged for invalidity.

### Institutionalization of bias and equity

According to [24], validity relies on outcome consequences. Failure to accurately classify a specific subpopulation can negatively impact interpretive validity when the assessment imposes an undue burden on that subpopulation. Misclassifying NNES students may create instances of academic misconduct, generate greater scrutiny from faculty, increase levels of psychological stress and anxiety, establish a poor reputation for the student, and prolong their progression through the educational institution. UNESCO guidance states that generative AI systems run a risk of favoring culturally dominant languages and values unless those systems are subject to bias audits and/or multilingual validation [28]. As part of the GAI-AEM prototype, all of the above-mentioned items are classified as Consequences, which are a function of how an AI-caused CIV affects future opportunity structure for NNES individuals in Tables 5 and 6.

**Table 5:** Experimental Evidence of AI Detection Bias

Validity Domain	NES Essays	NNES Essays	Validity Interpretation
Internal Structure	< 5% misclassification	> 50% Misclassification³	Differential Functioning and Introduction to CIV
Text Perplexity	High complexity = human	Low complexity = AI	Misinterpretations of Linguistic Simplifications
Score Stability	Stable Classification	Significant False Positive Rate	Reliability Threat
Consequences	Low Risk	High Risk of Misconduct Accusation	Consequential Validity Threat

**Table 6:** Comparative validity effect across language groups (NES and NNES) in AI-powered assessments

Features	NES Student	NNE Student	Validity Implication
AI identification	High accuracy	High false-positive rate	Tilting (or differential) measurement bias
Text complexity metrics	Accepted as genuine	Suspicious	CVI based on linguistic content norms
Automated scoring	Very stable	Potential for downward scoring	Scoring error (distortion)
Bias in training data	Minimum impact	Structural disadvantage	Need for multilingual validation

Table 5 Demonstrates a side-by-side comparison of how the same AI detection features produce radically different outcomes for NES versus NNES writers. The disparity in false-positive rates (over 50% for NNES vs. under 5% for NES) is not a subtle difference; it is a gross violation of measurement invariance. The validity interpretation column makes this explicit: what is measured shifts from authorship authenticity to conformity with standard English perplexity patterns. No amount of technical recalibration can fix this without retraining on diverse corpora

Studies in Table 6 extends the comparison from detection to scoring and training data bias. The downward scoring bias (0.5–1.2 SD) is particularly consequential because it directly affects grades and progression. Unlike false positives, which can be overturned on appeal, a scoring bias operates invisibly students receive lower scores without ever knowing why. This hidden bias is arguably more harmful than false accusations because it lacks an obvious redress mechanism.

### Policy and ethical considerations associated with validity theory

International governance frameworks have recognized the potential of GenAI technologies to perpetuate dominance in the language used globally unless those technologies are purposely created in an inclusive manner [21]. In addition to ensuring that valid, equitable, and non-discriminatory admissions requirements are established, the use of the following validity-centered safeguards can help to prevent embedded inequities from occurring within institutional structures [30]. The application of modern validity theory has determined that generative AI tools influence fairness and validity not only through accuracy metrics but also through the degree to which their constructs are accurately represented across multiple subgroups.

The statistical biases reported above carry real-world consequences. False-positive rates of 55.7% for NNES writers translate directly into risk of academic misconduct accusations [15], and survey data confirm that 79% of NNES students experience considerable anxiety about such accusations [27]. While no longitudinal studies have tracked whether false positives lead to differential progression rates or attrition, the combination of high statistical bias and documented student fear establishes a prima facie case for precautionary governance. The causal pathway from algorithmic bias to educational harm is plausible, evidence-supported at each intermediate step, and demands structural intervention regardless of currently missing long-term data.

#### RQ3

Content, response process, internal structure, relationships with other variables, and consequences are important for determining fairness and equity in assessment.

*(How students interpret and engage with GenAI in assessment contexts)*

### Opportunity and utility as perceived by students

Among 2,847 medical students across eight survey studies, 79% expressed considerable apprehension about using GenAI in high-stakes assessments. By contrast, only 12% of native speakers in the same studies reported similar concerns. This disparity suggests that NNES students perceive a fundamentally different risk benefit calculus, driven primarily by fear of false misconduct accusations rather than by actual cheating behaviour.

According to empirical data, medical students use GenAI tools significantly and widely. A multicenter study conducted in Canada found that approximately 79% of participating medical students utilized GenAI tools [18]. The benefits reported by students include synthesizing large amounts of complex medical material, checking and receiving guidelines, and receiving rapid formative feedback. The importance of GenAI for students from English as a second language is that it may reduce their cognitive load as it relates to linguistic issues.

### Construct representation and construct-irrelevant variance

Students worry that AI-produced feedback is not a valid representation of how well they know what they are doing, that it may have hidden biases, and/or that AI policies in academia may be more beneficial to some students than to others [36, 40]. If having an artificial intelligence (AI) tool enhances their writing ability (language) without changing their ability to apply clinical reasoning skills, then it allows them to demonstrate their knowledge more clearly through improved construct representation. If AI detection tools misclassify student writing or institutions use the term “linguistic polish” to mean “authenticated,” then a student’s ability to produce language fluently becomes a construct-irrelevant factor and compromises the validity of assessments. This competing view affects students’ understanding of fairness.

### Consequential validity

Consequential validity is the examination of the social and psychological consequences of assessment systems. The results of this dimension were overwhelmingly aligned with this dimension.

#### Perceived fairness

When asked to respond regarding Gen AI in assessments, students were divided with respect to their responses, with approximately half expressing concern regarding reliability and misinformation [18]. Moreover, they mentioned that the students are also afraid of facing punitive actions and thus do not feel that they have been treated fairly. For example, non-native speakers of English have mentioned their fears of being placed under unreasonable suspicion and falsely accused of cheating by using AI and language divergence. Therefore, the sense of fairness is relative to transparency and governance.

#### Ethical dilemma-induced anxiety

Many researchers have studied students’ confusion about their professional responsibilities and mistrust of others. The ambiguity surrounding policy guidance, the opacity of AI detection systems, and the perceived vulnerability of students who use a different language can all contribute to heightened anxiety levels within high-stakes environments where assessment is being performed. In terms of validity, the effect of anxiety may cause individuals to perform poorly, be unable to perform to the best of their abilities, and systematically disadvantage themselves. Therefore, the impact of emotional distress can be added to the impact of consequential validity [5, 41].

**Table 7:** Integrated conceptual alignment. The conceptual framework for this review can be articulated as follows: GenAI Systems → Assessment Mechanisms → Validity Domains → Student Outcomes as in Table 7

Framework Component	Validity Domain	RQ3 Implication
GenAI Systems AI-assisted feedback	Response Process	Potential reduction of linguistic load
GenAI Ai Detection Systems	Construct Validity	Possible construct-irrelevant variance due to poor use of AI detection systems
Institutional governance	Consequential validity	Determines fairness perception
Policy clarity	Consequential validity	Reduces anxiety
Inclusion of Linguistic Diversity	Content validity	Enhances equity

Table 7 Translates the conceptual framework into student-level outcomes. The key distinction is between GenAI as feedback and GenAI as detection. Students experience this tension directly, and their perception of fairness. Table 8 Depends almost entirely on which of these two functions dominates in their institution’s policy. The table therefore implies that institutional governance not the tool itself determines whether students experience GenAI as enabling or threatening.

**Table 8:** Thematic Coding (Student Perceptions of GenAI in High-Stakes Assessment)

Theme	Validity Domain	Perceived Impact	Equity Implication
Utility and effectiveness	Response process	Increased preparatory skills	Equity enhancement
Writing assistance	Construct validity	Minimizes the language load	May decrease the construct-irrelevant variance
Fear of detection	External validity	Increased anxiety	Risk of an inequitable consequences
Ambiguity in the policies	External validity	Perception of unfair advertising	Entitlement inequities
Distrust in AI	Response process	Hesitancy to use	Opportunity inequalities
Ethical issues	External validity	Professional identity conflict	Moral stress in a high-stakes settings

This Table 8 represents the thematic codes in division in student consciousness. On one hand, utility and writing assistance are valued. On the other, fear of detection, policy ambiguity, and distrust create anxiety and perceived unfairness. The critical finding is that the same student can hold both sets of beliefs simultaneously. This ambivalence means that simple survey questions about “attitudes toward AI” will miss the nuance; what matters is the balance between opportunity and threat, which varies with institutional transparency.

## Discussion

The findings of this review support three main claims. First, GenAI assessment tools produce systematic measurement bias against NNES medical students. Second, this bias arises from the enforcement of dominant linguistic norms embedded in training corpora. Third, NNES students perceive this bias acutely, with policy ambiguity magnifying their anxiety.

### Validity as interpretive justice

Validity theory, as applied today, does not see validity as an attribute of a test [16, 42] but rather as an attribute of the way the resulting scores are interpreted and used. Fairness and interpretive defensibility across subgroups are inseparable within this framework. This raises the question of whether GenAI produces accurate predictions, and to what extent does GenAI support defensible interpretations of assessment results for linguistically diverse students.

According to [15, 34] NNES writing in AI detection has indicated a high rate of false positives. With regard to this evidence, we can conclude that measurement invariance is impaired. While the use of algorithmic classifiers systematically associates linguistic predictability and artificial authorship, the association introduces CIV [43] is not relevant to CRC. Such distortions affect the internal structural evidence that is necessary to support the comparable interpretation of scores between groups. These errors extend beyond statistical anomalies in high-stakes situations.

### Linguistic normativity and construct representation

Furthermore, the findings imply that GenAI systems might replicate prevailing language standards due to the inclusion of these standards within their training datasets [19]. In assessment contexts, such normative systems call into question which members’ language behaviors are authenticated as legitimate displays of professional capabilities [44]. Despite the fact that medical education is being provided more frequently than ever to a global and multilingual student body, algorithmic systems may prioritize standard written academic English as the conventional reference point for what constitutes authenticity. The relationship between language clarity and language conventions creates tension from the perspective of representation construction. Clinical reasoning is different from rhetorical style or polish. However, automated systems that reward fluent speech patterns consistent with mainstream language norms provide NNES students with an opportunity to be evaluated based on their similarities to NES-like expressions rather than their clinical or ethical reasoning abilities [37, 45].

### Response processes and assessment ecology

The validity argument is made more complex because of the perceptions of students. Surveys have shown that many students are using GenAI to help them learn and prepare for assessments [46]. NNES students have indicated that receiving AI-assisted feedback reduces their cognitive load and clarifies their expression, which may help mitigate language-related cognitive invalidity. GenAI may also improve the constructive representation of learning because it allows for a more precise expression of reasoning [47]. However, students have reported that using such systems can also cause them to have anxiety due to the lack of a clear understanding of the policies that govern their use or how detection methods work [26], such an ambiguous situation creates a dual experience where GenAI is changing the way that students approach responding. Students’ engagement strategies may depend on their learning goals and their expectations regarding being surveilled or incorrectly classified. When assessments are performed in environments with ambiguous policies regarding AI use, some students may fail to use support tools to their fullest potential (or possibly over-correct) to avoid being perceived as suspicious. Such changes to how students use resources impact the evidence that connects performance and construct. The difficulty of drawing conclusions and generalizing language based on [42] framework arises when, in any of the cognitive response processes, we find that groups have different perceptions of risk. In this case, the comparability of test scores is jeopardized regardless of any overt scoring bias present in the dataset. Thus, fairness issues can arise from the algorithms and the larger ecosystem of computer-mediated assessment processes.

### Reframing equity in global medical education

As the medical training landscape continues to evolve into something that crosses borders, it is also creating a more linguistically diverse participant base. Therefore, any assessment process must accommodate multiple forms of expressions because linguistic variation is not an indicator of limitation. The age-old assumptions of language, authorship, and authenticity implicit in written assessment become more apparent by integrating GenAI into evaluation processes. The question is how to incorporate AI into evaluation systems without re-creating those existing linguistic hierarchies that have been established for years. Moving away from aggregate claims regarding accuracy and shifting to subgroup-sensitive validation can accomplish this task. Additionally, providing institutions with direction on designing assessment tools and processes that are, by nature, equitable as opposed to merely neutral or unbiased as it relates to technology can also help achieve equity in the assessment of participants regardless of their language.

### Considerations from broader educational research

While the present systematic review focused exclusively on medical students and assessment equity, recent studies in other educational contexts have raised complementary concerns about AI dependency and critical thinking. Studies examined preservice mathematics teachers’ adoption of AI chatbots, finding that higher AI literacy did not protect against overreliance; rather, the highest AI literacy profiles exhibited the greatest dependency [48, 49]. Their structural equation modeling revealed that dependency mediates the literacy-skill relationship, such that AI literacy’s direct effect on critical thinking is positive only when accompanied by critical engagement; otherwise, its indirect effect through dependency is negative [14, 50].

These findings from teacher education contexts suggest that the risks of AI dependency and reduced critical thinking may generalize beyond mathematics education to medical training. However, future research is needed to confirm whether similar mechanisms operate in medical education, where clinical reasoning and patient safety are at stake. For NNES medical students, these risks may compound with linguistic bias: the same AI tools that reduce language barriers may inadvertently encourage overreliance, while algorithmic bias in detection systems creates additional barriers.

### Recommendation for practice and policy

Give clear guidelines on the use of AI. The results that three out of five NNES students claim to have uncertain or missing institutional policies (among 2,847 surveyed students) are a changeable trigger of anxiety and injustice. All syllabuses must include i.e., a simple statement on what AI can be used, illustrations of how AI should be cited, and how to contest detection flags.

Separate language and content. The reduced bias in automated scoring NNES students (0.5–1.2 SD) indicates that most rubrics in use today lump linguistic polish and clinical reasoning. Two-domain rubrics (clinical reasoning 70–80, language clarity 20–30) and offering language supports to NNES students (e.g., grammar checkers, extended time, bilingual dictionaries) should be used by educators to guarantee that intended constructs are measured in assessments.

Make transparent AI governance and equity requirement. The fact that the problem of policy ambiguity correlates with both increased anxiety (79% of NNES students) and a sense of unfairness (63% of students referencing equity issues) shows that governance does not only rely on the administrative side but has a direct impact on student performance. Institutions must: (a) release an AI evaluation policy that is publicly available; (b) create an AI ethics advisory board, with student membership, including NNES students; (c) provide funds to audit bias and appeals.

## Conclusion

Synthesizing the evidence across 27 studies, we find that GenAI does not create entirely novel forms of assessment inequity but rather amplifies pre-existing construct-irrelevant variance related to linguistic background. Evidence for this claim includes: (a) AI detection systems penalize linguistic simplicity (a known feature of NNES writing) at rates > 50%, whereas native-speaker writing is rarely misclassified (< 5%); (b) automated scoring systems show systematic downward bias for NNES writers (0.5–1.2 SD); and (c) student perceptions indicate that policy ambiguity, not the technology per se, drives fairness concerns. These patterns reflect long-standing challenges of linguistic normativity in assessment, now algorithmically mediated.

The impact of GenAI on medical education assessment can be framed through the lens of validity (including a focus on the consideration of interpretive justice among communities/language groups). For non-native English speakers (NNES) in medical school, GenAI simultaneously represents opportunity, risk, and ambiguity (creating greater or lesser degrees of inequity). Whether GenAI reduces or increases inequity depends on how well the systems are designed and evaluated with explicit consideration for the validity of subgroups and constructs. Thus, GenAI raises no new challenges to equity; rather, it enhances existing challenges to equity in terms of language proficiency and competence inference.

### Limitation of the study

The absence of longitudinal studies means we cannot determine whether GenAI use leads to genuine improvement in NNES students’ unassisted English proficiency or creates long-term dependency. All included studies measured short-term performance or perceptions only. Future research should track linguistic development and autonomous writing ability over at least one academic year. English-language publications were considered only, and it is possible to have missed some studies in other languages. Included studies are heavily skewed towards English-dominant countries (Fig. 4), which restricts the generalizability of the results to non-Western educational settings that consider English as a second language, not a first language.

The time frame for the literature review (2022–2025) covers the rapid growth of GenAI since the launch of ChatGPT at the end of 2022. Nonetheless, due to the short window of time, the body of research is still in its infancy. A majority of the selected papers (*n* = 11) are cross-sectional surveys, while others (*n* = 6) utilize experimental study designs.

The 78% of reviewed studies belong to the countries where English is the native language (USA, Canada, Australia and UAE). Evidence is deficient in Sub-Saharan Africa, South Asia, Latin America, Europe where the English is not native. This biased representation suggests that the findings cannot be generalized to any other situation assuming that (a) students and tutors are not fluent in English; (b) all participants are second or third language speakers; or (c) the rules and methods of evaluation of academic integrity are not similar.

The quality appraisal showed that 7 (25.9) studies only satisfied 60–79% of MMAT/AXIS criteria and 2 (7.4) studies only satisfied less than 60%. Common weaknesses were single-institution samples, unvalidated instruments, not controlling for confounding (e.g., previous academic performance and English proficiency scores) and small sample sizes in cases of subgroup analysis. These lower-quality studies are needed to be incorporated in the synthesis, and can either over- or under-estimate actual effect sizes.

### Future research

Further research should include experimental comparisons of assessment results using AI-assisted assessments between English language students who are native to the English language and those who have other languages as their mother language. These studies must have specific disaggregation of misclassification, rates, degree of automated scoring inconsistency, and disciplinary outcome.

Longitudinal cohort studies are needed to assess whether NNES students who use GenAI tools show measurable gains in unassisted English writing proficiency over time, or whether reliance on AI inhibits autonomous language development.

## Data Availability

This study used secondary data therefore, no dataset was used or generated. The data used for systematic review is able can be submitted on request with the acceptance from all the co-authors.

## Abbreviations

NNES: Non-native English-speaking
CIV: Construct-irrelevant variance
NES: Native English speakers

## Glossary

Internal structure: Consistency of measurement across subgroups
Construct validity: Extent to which a test measures the intended construct
Consequential validity: Social and psychological consequences of score use
Construct‑irrelevant variance: Variance due to factors unrelated to the target construct
